# Mesenchymal Stem Cells Migration Homing and Tracking

**DOI:** 10.1155/2013/130763

**Published:** 2013-09-30

**Authors:** Abhishek Sohni, Catherine M. Verfaillie

**Affiliations:** Stem Cell Biology and Embryology, Department of Development and Regeneration, KU Leuven, 3000 Leuven, Belgium

## Abstract

In this review, we discuss the migration and homing ability of mesenchymal stem cells (MSCs) and MSC-like cells and factors influencing this. We also discuss studies related to the mechanism of migration and homing and the approaches undertaken to enhance it. Finally, we describe the different methods available and frequently used to track and identify the injected cells *in vivo*.

## 1. Potential of MSC and MSC-Like Cells for Cell-Based Therapies

For stem cells to be used in the clinical setting, they should be safe, that is, do not form tumors, and be readily harvested and/or expanded. Although embryonic stem cells (ESCs) are pluripotent and could be used to replace any tissue, they can form teratomas. Hence, their potential use in cell-based therapies will require that no undifferentiated ESCs persist in the graft. In addition, culture methods for human ESCs are still quite demanding; hence, scaleup is not yet straightforward.

The best-studied transplanted stem cell is the hematopoietic stem cell (HSC) that can be harvested from different sources (bone marrow, blood, and umbilical cord blood) in sufficient numbers for transplantation. HSCs have then also been used for cell-based therapies especially in an allogeneic setting for more than a quarter of a century.

Other adult stem cell populations that are being evaluated clinically are MSCs and multipotent adult progenitor cells (MAPCs; trade name MultiStem) both derived from human postnatal tissue.

MSCs were first described in the 1970s by Friedenstein et al. who described a population of cells derived from bone marrow that had the appearance of fibroblasts and could generate aside from fibroblasts, also adipocytes, chondrocytes, and osteocytes [1, 2]. These cells grew out as colonies and were therefore termed “colony forming units” or CFUs. Later on, Caplan and others termed these cells “mesenchymal stem cells” (MSCs) [3, 4]. MSCs are classically isolated from bone marrow however; they can be found in multiple tissues, such as adipose tissue, fetal lung, placenta, Wharton's jelly, and UCB, among others [5–8]. MSCs are characterized as adherent cells with the ability to differentiate into fibroblasts, adipocytes, chondrocytes, osteocytes, and smooth muscle cells apart from supportive hematopoietic “stromal” cells [1, 3, 4, 9] and with a characteristic cell surface antigen profile.

Many MSCs or MSC-like cells with varying differentiation potential have been described and reviewed elsewhere [10]. Although the cell surface repertoire and the gene expression pattern vary among these cells, this is likely a reflection of the tissue of origin or the culture conditions used for maintenance of these cells [11, 12]. A standardized phenotype was proposed for MSCs by the International Society for Cellular Therapies. A typical human (h)MSC should express CD105, CD90, and CD73 but not CD79a, CD45, CD34, CD19, CD14, CD11b, and HLA-DR on its surface [3, 4, 13]. Most hMSCs or hMSC-like adult progenitors give rise to mesoderm derivatives such as fat, bone, and cartilage [9]. Apart from the mesenchymal lineages, MSCs and MSC-like cells such as hMAPC have been reported to also be able to give rise to skeletal myocytes, cardiomyocytes smooth muscle cells, and endothelial cells [11, 14–16] (also reviewed in [10]). Although some studies have suggested that MSCs can give rise to neurons and endodermal progeny [17–19], it remains unclear whether such progeny has all properties of primary neuroectodermal and endodermal cells.

Apart from being able to differentiate to multiple cell types *in vitro* (and *in vivo*), MSCs and MSC-like adult stem cells have extensive immunomodulatory and immunological tolerance inducing characteristics [20–26]. hMSCs that characteristically lack expression of MHC-II, CD40, CD80 and CD86 but express MHC-I present themselves as nonimmunogenic. Although the presence of MHC-I may activate T-cells, due to lack of costimulatory molecules, MSCs fail to elicit an immune response [27]. MSCs also efficiently suppress an immune response by modulating T-cell activation and proliferation [28, 29], either by a direct cell-cell interaction [30] or mediated via soluble factors [28, 31] and this is independent of MHC matching. This immunomodulating effect of MSCs is being explored as adjuvants during allogenic transplantation to prevent graft-versus-host disease (GVHD) [32, 33] and during organ transplantation to prevent immune rejection [29, 34–36]. In addition, the immunomodulatory characteristics of MSCs are being evaluated in the setting of autoimmune diseases, such as Crohn's Disease, among others [37, 38].

MSCs also produce innumerable growth factors and cytokines, which make them suitable for inducing endogenous repair. For instance, MSCs express bone morphogenic protein(s) (BMPs) which is effective in enhancing cartilage, bone, and tendon repair [39]. Likewise, MSCs produce factors that enhance revascularisation, even if their nature is not understood, and are therefore being evaluated in therapies for ischemic disorders, such as stroke, myocardial infarct, or peripheral arterial disorders. Yet another field of therapeutic applications is grafting MSCs that have been genetically modified to overexpress a protein in diseased tissues due to the genetic mutation of the given factor.

## 2. Homing of MSCs

The lingering problem in the field of cell-based therapies is the delivery of the cells to the site of injury, a process termed “homing.” As discussed above, the therapeutic efficacy of MSCs is greatly dependent on their ability to produce juxtacrine or paracrine factors that enhance regeneration from endogenous (stem) cells. For juxtacrine effects to be possible, migration of MSCs to the diseased organ/tissue is required. Migration and homing to the tissue of injury is influenced by multiple factors including age and passage number of the cells, culture conditions, and the delivery method, among others. We here provide a review of the literature demonstrating the effect of various factors on migration and homing of MSCs.

### 2.1. Age, Passage Number, and Dosage of MSCs

It has been shown that with higher passage number, the engraftment efficiency of MSCs decreased. Rombouts et al. had performed a time course experiment, where they showed that freshly isolated MSCs had a better efficiency of homing compared to cultured cells [40]. Moreover, they showed that culture of MSCs for 24 hr decreased the homing efficiency to 10% from 55–65% and to near 0% when cultured for 48 hr. It is well documented that with age, the ability of an organism to repair and heal goes down which is in part due to decreased potency of resident stem/progenitor cell. Thus, it is possible that *in vitro* multiplication also causes “aging” and hence decreases potency. However, another possibility is that for other stem cells like HSCs, culture alters the expression and function of cell surface ligands required for homing; it will be discussed below.

### 2.2. Source and Culture Conditions of MSCs

As alluded earlier, MSCs can and have been isolated from multiple different tissues [41] with differences in the phenotype of the cells isolated [42]. These differences are likely in part due to differences in the native microenvironment from where they are isolated [43]. This presents a challenge for the use of MSCs for therapeutic purposes. In order to define an MSC, the Mesenchymal and Tissue Stem Cell Committee of the International Society for Cellular Therapy (ISCT) proposed certain standards to be considered while using human MSCs therapeutically [44]. Apart from the source of the MSCs, culture methods greatly influence MSC characteristics, including their homing potential. As mentioned earlier, freshly isolated MSCs home better than their cultured counterparts [40]. The CXCR4 chemokine receptor that recognizes CXCL12 (also termed SDF-1*α*) is highly expressed on bone marrow MSCs, but is lost upon culturing [45, 46]. However, when MSCs are cultured with cytokines (such as HGF, SCF, IL-3, and IL-6) [47], and under hypoxic conditions, CXCR4 expression can be reestablished [48]. Similarly, matrix metalloproteases (MMPs), known to be important in migration of cells, have been demonstrated to play a role in MSC migration [49–51]. Expression of MMPs in MSCs is influenced by factors such as hypoxia [50] and increased culture confluence [49]. Moreover, inflammatory cytokines TGF-*β*1, IL-1*β*, and TNF-*α* also enhance migration by upregulation of MMPs (MMPs) [51] affecting homing of MSCs. Hence, culture conditions to which MSCs are exposed play a vital role in their homing ability.

### 2.3. Delivery Method

The efficacy, bioavailability, and functionality of a pharmacological drug are dependent on the method via which it is being administered. In order to enhance efficacy and availability, the method of administration of MSCs should hence facilitate homing of MSCs to the desired tissue. Intravenous infusion is one of the major routes of administration of MSC [52–55]. When MSCs are infused systemically, they are trapped into capillary beds of various tissues, especially the lungs [52, 56–58]. Therefore, intra-arterial injection of MSCs has been assessed. Delivery of MSCs via the internal carotid artery significantly improved their migration and homing in the injured brain compared with injection via the femoral vein [59]. Similarly, in humans with subacute spinal cord injury (SCI), delivery of MSCs via the vertebralis artery leads to a greater functional improvement than when cells were administered via the intravenous route [60]. However, delivery of cells in an artery may lead to “microvascular occlusions” [59]. While to treat myocardial infraction (MI), delivery of bone marrow cells or MSCs directly in the heart or close to the site of injury enhances the number of cells found in the peri-infarct region [61]. Similarly, direct injection of adipose-derived MSC in damaged skeletal muscle leads to an increase in mass and functional capacity [62].

### 2.4. Host Receptability-Injury versus Noninjured

MSCs have the luxury of being tolerated by the host immune system due to low immunogenicity as discussed earlier. Their bioavailability and efficacy are dependent on the host pathological condition. During an injury, host cells release different chemo-attractants that have a positive influence on homing of MSCs. This possibly explains the observation that MSCs home better when injected 24 hrs after injury than after 14 days in a myocardial injury model [63]. Many such chemoattractants and the associated receptors on MSCs have been identified. Moreover, MSCs are being genetically engineered to overexpress such receptors to enhance their homing to the damaged tissue [61, 63–66]. Moreover, strategies to precondition the host for better distribution and to prevent injected cells from being entrapped in small vessels especially of the lungs have proven beneficial. One such approach was the pretreatment of host with vasodilator such as sodium nitroprusside (SNP) which resulted in increased MSC passage through the lung microvasculature compared to untreated hosts [58].

## 3. Mechanism of Homing

Most insights in the mechanisms underlying migration and homing are from studies that evaluated leukocyte migration [67] into inflamed tissues, HSCs [68] the and metastatic cancer cells [69]. A significant body of the literature also exists related to mechanism of MSCs migration towards the target tissue and the role of cell surface receptors and molecules in aiding this migration. The role of activated endothelial cells in migration of MSCs is also being extensively studied. We here describe the factors that aid MSCs in migration and homing to tissue of interest.

### 3.1. Expression of Receptors and Adhesion Molecules

Similar to leucocytes, MSCs express many receptors and cell adhesion molecules that aid in migration and homing to target tissues. However, the precise mechanisms by which MSCs are recruited are not yet fully understood.

Homing is in a significant part dependent on the chemokine receptor, CXCR4, and its binding partner that was previously characterized in HSC homing, that is, stromal-derived factor-1 CXCL12 [61, 64, 70–72]. Wynn et al. demonstrated that CXCR4 is resent on a subpopulation of MSCs, which aid in CXCL12-dependent migration and homing [45]. Aside from CXCR4, freshly isolated BM MSCs and cultured MSCs also express CCR1, CCR4, CCR7, CCR10, CCR9, CXCR5, and CXCR6 [72, 73] which are also involved in MSC migration

Integrins are another family of cell surface molecules involved in migration of variety of cells and are expressed on adipose-derived MSC-like cells [74]. Neutralizing antibodies against integrins, more specifically the integrin-beta1 integrin, but not integrin-alpha4, inhibit, MSC homing to infracted myocardium [75]. However, other studies have shown that integrin-alpha4 plays a role in MSC migration [76]. Interestingly, integrin ligands such as VCAM and ICAM are also expressed on MSCs [77].

### 3.2. Interaction with Endothelial Cells

Migration and homing requires that cells can attach to and migrate between endothelial cells (ECs) to enter the target tissue. While it well established that leukocytes attach to ECs, roll over the ECs, and then transmigrate between ECs, how MSCs interact with ECs is not well understood. MSCs express molecules as a number of adhesion molecules, including selectins and integrins, involved in these steps. Rüster et al. using a parallel plate flow chamber, demonstrated that MSCs like HSCs bind to ECs derived from human umbilical cord vein (HUVECs) [76]. The binding was enhanced when ECs were activated by TNF-*α* [76]. The cells migrate by extending podia followed by rolling and adhesion on the EC. They further demonstrated that the binding and rolling of MSCs were mediated by the P-selectin adhesion molecule, whereas migration involved the binding of VLA-4 (or integrin-beta1 & integrin-alpha4 dimer) on MSC with VCAM-1 found on ECs [76]. Steingen et al. found a similar mechanism by which VLA-4/VCAM-1 is required for transendothelial migration. In addition, migration was dependent on the phenotype of the vascular bed [78] and also involved proteolytic enzymes [78]. This is consistent with the studies from De Becker et al. and others demonstrating a role of the MMP class of proteolytic enzymes in MSC homing and migration [49, 51]. MMP-2 belongs to the gelatinase class of proteolytic enzymes that cleave gelatin and collagen-IV, the two major constituents of basement membrane.

## 4. Approaches to Improve Homing

For MSCs to home and target a specific tissue, they require the right combination of signaling molecules from the injured tissue and the corresponding receptors on MSC. The expression of chemokine receptors on MSCs is influenced by many factors. Although freshly isolated MSCs home better, only limited numbers of cells can be isolated. Therefore, approaches to expand MSCs while retaining expression of receptors needed for efficient homing are being developed. For instance, pretreatment of cultured MSCs with cytokines (such as IL-6, HGF, etc.) increased expression of chemokine receptors (CXCR4) and improved their migration both *in vivo* [47] and *in vitro* [79]. Likewise, IL-1*β* pre-treatment enhanced the efficacy by MSCs homing in a colitis model [80].

Other approaches include changing culture conditions and coculture of MSC. Hung et al. demonstrated that short-term exposure of MSCs to hypoxia leads to increased expression of chemokine receptors (CX3CR1 and CXCR4) that may aid engraftment *in vivo* [81]. A similar increase in chemokine receptor (CXCR4) apart from cell proliferation-associated cyclin (cyclin D1, D3) expression was observed when humans umbilical cord MSCs were cocultured with Sertoli cells [82]. Yet another approach is the use of genetically engineered MSCs that overexpress chemokine receptors such as CXCR4 and integrin-alpha4 to influence their homing ability. Kumar et al. transduced MSCs with an adenovirus encoding integrin-alpha4, which enhanced their ability to home to bone [83]. A similar approach was taken to overexpress CXCR4 in MSCs to enhance their homing ability and to improve recovery after myocardial infraction [84]. Compared with untransduced MSCs, CXCR4 overexpressing MSCs resulted in a decrease in anterior wall thinning, and left ventricular chamber dimensions were better maintained and remodeling was observed [84]. Although these genetically modified MSCs may not yet be available for therapeutic use in humans, pre-treatment approach may well be applicable.

## 5. Tracking Mesenchymal Stem Cells *In Vivo*

As homing of MSCs is inefficient and many MSCs are trapped in the lung following systemic administration, it is imperative that we can trace the fate of the injected cells. One classical method to label cells is with retroviral vectors to express fluorescent proteins, which has been helpful in gaining insights in MSC homing and engraftment [85]. However, the visualization of cells that homed in different organs requires sacrificing of the animal, as the tissue penetrability of fluorescence is limited. Hence, more advanced techniques to track the injected cells *in vivo*, such as bioluminescence imaging (BLI), single-photon emission CT (SPECT), positron emission tomography (PET), multiple photon microscopy, and magnetic resonance imaging (MRI), are being employed. The noninvasive cellular imaging allows for tracking the injected cells in multiple tissues and over time.

To trace cells by MRI requires labeling of the cells with contrast reagents in order for the cells to be visualized. Cells can be labeled with contrast agents: either positive contrast agents used in T_1_-weighted MRI such as lanthanide chelates [86] or Mn-containing compounds [87, 88]; or negative contrast agents, such as superparamagnetic iron oxide (SPIO) [89–92], ultra-small superparamagnetic iron oxide (USPIO) particles [90, 92, 93], or micron-sized iron oxide particles [94, 95], that are highly sensitive and have a dominant effect on the T_2_/T_2_* relaxation times, causing negative contrast enhancement in the regions of interest. For use with stem cells, the role of these agents on cell potency and function needs to be evaluated. Crabbe et al. evaluated the effect of different MRI contrast reagents on the cellular function of embryonic and postnatal stem cells including MSC in an ischemic stroke model [96]. Differences were observed in terms of size, densities, and number of inclusions among the different reagents tested. Moreover, the labeling did not interfere with the migratory capacities of these cells *in vivo *[96]. One drawback of MRI-based cell tracking is that whole body scans are difficult to achieve, and, hence, determination of where the labeled cells traffic to other than the tissue that is damaged, is difficult. A second drawback is that the iron oxide particles are retained in a tissue even if the grafted (stem) cell dies, hence, leading to false positive signals. Yet another problem as shown by Vandeputte et al. is that a pronounced hypointense signal intensity on 3D T_2_*w MR images can be seen spontaneously, corresponding to damaged blood vessels and inflammatory cells, thus, warranting caution [97].

PET imaging relies on the activation of a tracer dye by a protein such as herpes simplex virus type 1 thymidine kinase (HSV1-tk) or varicella zoster virus thymidine kinase (VZV-tk), expressed by genetically engineering into the injected cells [98, 99]. Two main tracer classes used as probes for HSV1-tk substrate are pyrimidine nucleoside derivatives and acycloguanosine derivatives [100]. PET/SPECT visualizes the emission from a tracer dye that can be toxic and interferes with cellular function. Alternatively, cells can be directly labeled with tracer dyes such as ^18^F-FDG. Wolfs et al. demonstrated that ^18^F-FDG, a positron emitting glucose analogue, can be easily taken up by MSC and MSC-like cells without interfering with cellular functions [101]. Further, the presence of this analogue did not significantly affect the viability, proliferation, differentiation, and migratory capacities of these cells [101]. However, this only allows following the cells for a short period of time, as the tracer decays over 109 minutes [102].

BLI requires the expression of a bioluminescence protein that can be visualized and is hindered less by tissue mass unlike fluorescent proteins. With the availability of instrumentation for visualization, the BLI method has been quite useful in tracing cells in mouse models. Bioluminescence can be obtained using proteins from the luciferase family (firefly or *Renilla*), which again needs to be genetically engineered in the injected MSCs [103, 104]. In a study evaluating the immunogenicity of such xenoproteins in genetically engineered MSCs, Bergwerf et al. found no immunological response when such MSCs were injected in the brain. In contrast, they found reporter gene-specific immune-reactive T-cell responses when they were injected in the muscle [105].

Kraitchman et al., using dual labeled cells (tracer and contrast reagent), were able to follow the cells for up to a week in an MI mouse model [106]. Their tracing showed that the cells initially home to the lungs followed by redistribution to nontarget organs within 48 hrs. MSCs were also found at the site of infarct up to one week after injection [106]. However, the cells were unable to be located using MRI, rather, they were traceable using high sensitive SPECT [106].

Many such reagents and cell tracing methodologies have been developed and evaluated for stem and progenitor cells. The efficacy, toxicity and resolution are the main factors that determine the choice of imaging technique. 

## 6. Conclusion

Postnatal MSC or MSC-like cells are currently the primary source of stem cells that have found clinical relevance. Embryo-derived stem cells such as ESC although have a greater differentiation potential, they suffer from their ability to induce teratomas *in vivo*; hence, it has been difficult to translate their clinical use. As alluded here, a great deal of work has been done to harness the potential of these adult stem cells for the treatment of patients. MSC and the likes are already undergoing clinical trials for use in patients especially for their immunomodulatory features. However, their heterogeneity and off-target homing especially lodging in the lungs impede the clinical use of MSC and MSC-like cells. Owing to this, a large number of cells are required to obtain desired effect at the target organ(s). Different methods as discussed above, such as targeted delivery, cytokine pre-treatment, and assisted homing, are being used to circumvent such impedances.
